# S-Shaped Canals: A Series of Cases Performed by Four Specialists around the World

**DOI:** 10.1155/2014/359438

**Published:** 2014-07-16

**Authors:** Ricardo Machado, Antonis Chaniottis, Jorge Vera, Carlos Saucedo, Luiz Pascoal Vansan, Emmanuel João Nogueira Leal Silva

**Affiliations:** ^1^University of São Paulo, Ribeirão Preto, Brazil; ^2^Private Practice Limited to Endodontics, Kalithea, Greece; ^3^University of Tlaxcala, Puebla, Mexico; ^4^Private Practice Limited to Endodontics, Monterrey, Mexico; ^5^Grande Rio University, Rio de Janeiro, Brazil

## Abstract

Recognition of anatomical variations is a real challenge for clinicians undertaking therapy regardless of the teeth that are to be treated. The extent of the curvature is one of the most important variables that could lead to instrument fracture. In clinical conditions, two curves can be present in the same root canal trajectory. This type of geometry is denoted as the “S” shape, and it is a challenging condition. This report describes a different clinical and educational scenario where four specialists around the world present different approaches for the treatment of root canals with double curvatures or S-shaped canals. Endodontic therapy is a very nuanced and challenging science and art. The clinical and teaching experience of the authors show different approaches that can be successfully employed to treat challenging teeth having roots with multiple curves. The necessity of precise knowledge of the root canal morphology and its variation is also underlined.

## 1. Introduction

Straight simple root canal systems are exceptions and not rules in the human dentition. Nature frequently demonstrates curved root canal systems of high complexity with multiple curves in different planes [1]. Endodontic cleaning and shaping are difficult when such systems are presented [1–3]. Recent studies have highlighted the complexity of the root canal system [4–6], which can create significant endodontic treatment difficulties. Curves in multiple spatial orientations provide examples of these clinical challenges [1, 7, 8] and an “ideal” preparation can be a difficult task to achieve, especially in canals with these features [9, 10].

The aim of this paper is to show four cases of S-shaped canals performed with different approaches by four specialists from different clinical scenarios.

## 2. Case Reports

### 2.1. Case 1

**Figure 1:**
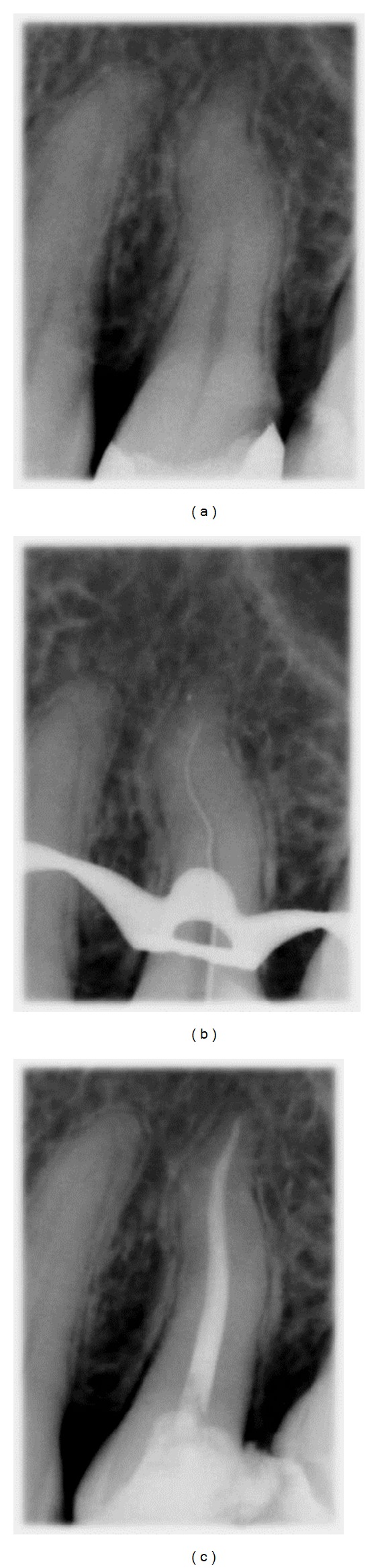
(a) Initial radiograph, (b) working length radiograph, and (c) final radiograph.

A 40-year-old male patient was referred to the clinic of one of the authors (Jorge Vera) with severe pain to cold stimuli in his upper left maxillary arch. The medical history was noncontributory. All teeth in the area responded within normal limits to thermal cold tests except for the second left maxillary bicuspid. Probing depths were within 3 mm for all teeth of the region. Preoperative radiograph revealed a distal decay in the second left maxillary bicuspid and a double curve or s-shaped anatomy. After considering all findings, a diagnosis of irreversible pulpitis was made (Figure 1(a)).

After administrating infiltration anesthesia (articaine 1 : 100.000 epinephrine), the rubber dam was placed and the access cavity preparation was performed with size 2 round burs (KG Sorensen Zenith Dental Aps, Agerskov-Denamark). Sizes .10 K and .08 K files (Dentsply Maillefer, Ballaigues, Switzerland) were initially used with the Slick Gel Lubricant (SybronEndo, Orange County, CA) to try to reach working length. The files initially reached a very short length, so a step-back procedure using 360° counterclockwise movement of each file was performed using K files sizes .15, .20, .25, and .30 (Dentsply Maillefer, Ballaigues, Switzerland) with slight apical pressure. At the completion of the use of the large-sized files, 5.25% NaOCl was irrigated into the root canal preparation and a size .10 K file was taken to working length as confirmed by the Elements Diagnostic Apex Locator (SybronEndo, Orange County, CA) and a check radiograph (Figure 1(b)). The .10 and .15 K files were used at length then a Crown-Down instrumentation technique [11] was performed using the sizes .25/.10 and .25/.08 twisted files (SybronEndo, Orange County, CA) to instrument the cervical and middle thirds of the preparation. The apical third was shaped with sizes .25/.06, .30/.06, and .35/.06 TF instruments. 5.25% sodium hypochlorite was used to irrigate the root canal system between every instrument and patency was maintained with a .10 K file throughout the cleaning and shaping procedure. Passive ultrasonic irrigation was performed with an Irrisafe ultrasonic tip (Satelec, Merignac, France) for 1 minute with the canal completely flooded with 5.25% NaOCl; the canal was then irrigated with 17% EDTA, dried, and filled with gutta percha and Kerr Pulp Canal Sealer (Kerr Corporation, Orange, CA) employing the Continuous Wave of Condensation Technique [12] using the Elements Obturation Device (SybronEndo, Orange County, CA). A down pack motion was performed to fill the apical 4 millimeters of the root canal and the remainder of the gutta percha was injected with the gutta percha extruder (Figure 1(c)).

### 2.2. Case 2

**Figure 2:**
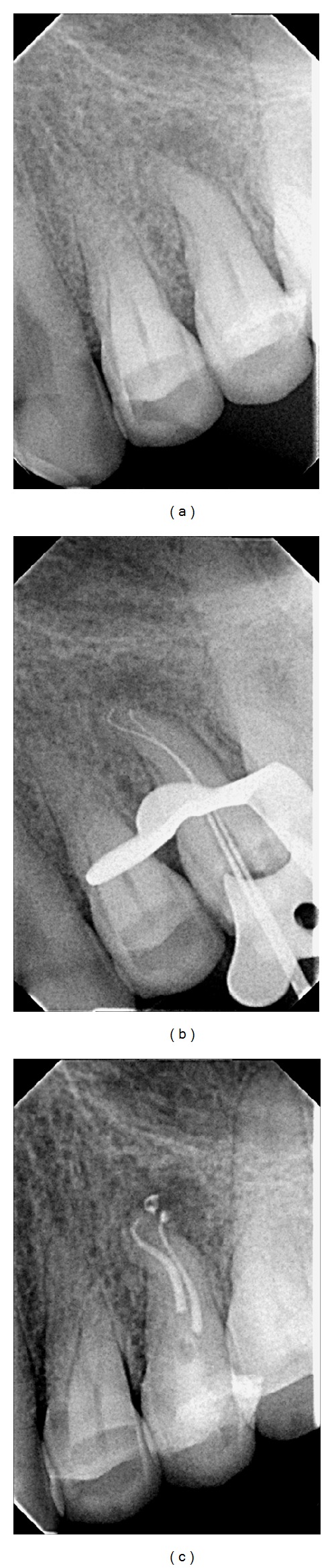
(a) Initial radiograph showing periapical radiolucency, (b) radiographic confirmation of the working length, and (c) immediate posttreatment radiograph.

A 60-year-old female patient was referred to the clinic of one of the authors (Antonis Chaniottis) for the evaluation and possible treatment of her left maxillary second premolar. The tooth was sensitive to palpation and percussion. The thermal and electrical pulp testing were negative. Thorough examination of the preoperative radiograph revealed a periapical lesion associated with the apex of the referred tooth and a double curve or S-shaped anatomy (Figure 2(a)). After considering all findings, a diagnosis of symptomatic apical periodontitis was unequivocally made.

After administrating infiltration anesthesia (articaine 1 : 100.000 epinephrine), the rubber dam was placed. Access was achieved by using the size 2 Endo Access bur (Dentsply Maillefer, Ballaigues, Switzerland). Refinement of the access cavity was achieved using the Endo Z bur (Dentsply Maillefer, Ballaigues, Switzerland). Coronal flaring of the canals was performed by using the Protaper SX rotary file (Dentsply Maillefer, Ballaigues, Switzerland).

The length determination radiograph revealed S-curve apical anatomy (Figure 2(b)). The initial negotiation and scouting of the S-curved canals were achieved with sizes .06, .08, and .10 K stainless steel hand files (Dentsply Maillefer, Ballaigues, Switzerland). The working length was verified using the Root ZX apex locator (J. Morita Inc., Kyoto, Japan) and confirmed radiographically (Figure 2(b)). Hand-filing was achieved by slowly inserting the K files (Dentsply Maillefer, Ballaigues, Switzerland) to the working length followed by gentle passive strokes upon withdrawal. This facilitated an unobstructed glide path to be created along the S-curve with minimal transportation during shaping.

After hand-filing, the sizes 1 and 2 Pathfinder rotary files (Dentsply Maillefer, Ballaigues, Switzerland) were used to working length, followed by scouting with sizes 10/.04 and 10/.06 Race files (FKG Dentaire, La Chaux-de-Fonds, Switzerland) to working length. No further enlargement of the S-curved canals was performed. 6% NaOCl was used to irrigate between each file used. Canal blocking was prevented by using multiple recapitulations with a precurved .08 stainless steel K files (Dentsply Maillefer, Ballaigues, Switzerland) between each rotary file use.

The irrigation efficacy was enhanced after completion of the shaping procedures by passive ultrasonic activation of the irrigant with a size .15 ultrasonic K file (Satelec Acteon Group, Merignac Cedex, France). The canals were next flooded with 17% EDTA solution for 2 minutes followed by a final rinse of sterile water. The canals were dried with size .20 sterile paper points and obturation was performed with the Continuous Wave of Condensation Technique [12].

Two fine feathered tip gutta percha points (SybronEndo, Orange, CA, EUA) were gauged to .20 and fitted with AH Plus sealer (Dentsply DeTrey, Konstanz, Germany) to working length. An extra fine tip mounted on the Elements Obturation unit (SybronEndo, Orange County, CA) was used at a setting of 200°C 5 mm short of the working length. The apical gutta percha was compacted by using a size 35 Dovgan plugger (G. Hartzell & Son, Concord, CA). Backfilling was performed using high-speed injection of thermoplasticized gutta percha by the Extruder Elements Unit (SybronEndo, Orange County, CA) through a .25 gauge needle (Figure 2(c)).

### 2.3. Case 3

A 32-year-old female patient was referred the clinic of the one of the authors (Ricardo Machado) with severe pain to cold stimuli in her upper left maxillary arch. The medical history was noncontributory. All teeth in the area responded within normal limits to the thermal and electrical pulp testing unless the left maxillary first premolar that showed a considerable hypersensitivity. Probing depths were within 3 mm for all teeth of the region. Preoperative radiograph revealed the presence of decay all around the crown and a double curve or s-shaped anatomy (Figure 4(a)). After considering all findings, a diagnosis of irreversible pulpitis was made.

After administrating infiltration anesthesia (articaine 1 : 100.000 epinephrine), the rubber dam was placed. Initial access was achieved by using a 1016HL bur (Dentsply Maillefer, Ballaigues, Switzerland) and refinement of the access cavity was achieved using the Endo Z bur (Dentsply Maillefer, Ballaigues, Switzerland). Coronal flaring of the canals was achieved by using the Protaper SX, S1, and S2 rotary files (Dentsply Maillefer, Ballaigues, Switzerland).

Initial negotiation and scouting of the S-curved canals were achieved with a size .10 stainless steel K file (Dentsply Maillefer, Ballaigues, Switzerland). Working length was verified by using the Elements Diagnostic Apex Locator (SybronEndo, Orange, CA, EUA). Hand-filing was achieved by slowly inserting the K files to the working length followed by passive gentle, withdrawal strokes. This allowed an unobstructed glide path to be developed along the S-shaped curvature with minimal transportation.

After hand-filing, a Crown-Down instrumentation technique [13] was performed by using size .04 through .30 profiles (Dentsply Maillefer, Ballaigues, Switzerland). A syringe of 2.5% NaOCl was used to irrigate the canals between each file use. Blocking of the canal was prevented by using multiple recapitulations with a size .10 K file (Dentsply Maillefer, Ballaigues, Switzerland) between each rotary file use. No further enlargement of the S-curved canals was performed. The canals were flooded with 17% EDTA solution for 3 minutes and dried with number 30 sterile paper points and the obturation was performed by the Tagger Hybrid Technique [14].

**Figure 3:**
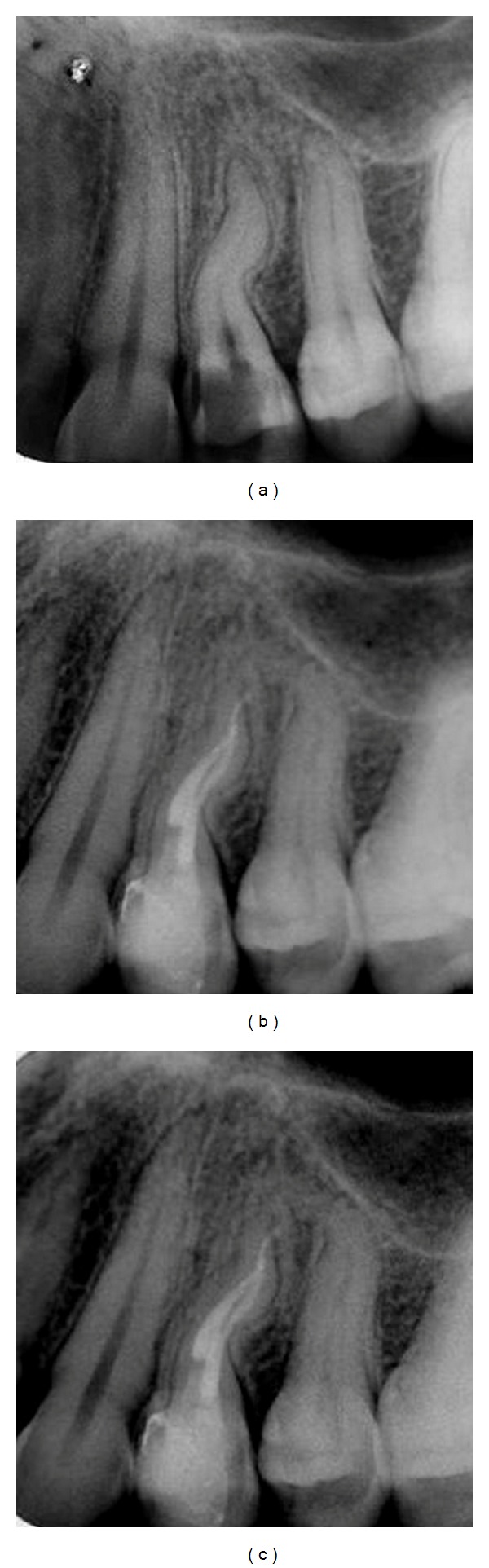
(a) Initial radiograph, (b) final periapical radiograph (straight view), and (c) final radiograph (mesial view).

Two gutta percha master cones (Profile .04—Dentsply Maillefer, Ballaigues, Switzerland) were fit to the radiographic terminus with firm tug back. The cones were coated with AH Plus sealer (Dentsply DeTrey, Konstanz, Germany) and fit to working length with the aid of a size 30 finger spreader (Dentsply Maillefer, Ballaigues, Switzerland). Subsequently, three accessory cones were added. Next a size 40/.02 McSpadden condenser was used limited to placement in the coronal two-thirds of the root. The tooth was temporized with Cavit (ESPE, Seefeld Oberb, Germany) and the patient was referred back to the referring dentist for the definitive restoration (Figures 3(b) and 3(c)).

### 2.4. Case 4

**Figure 4:**
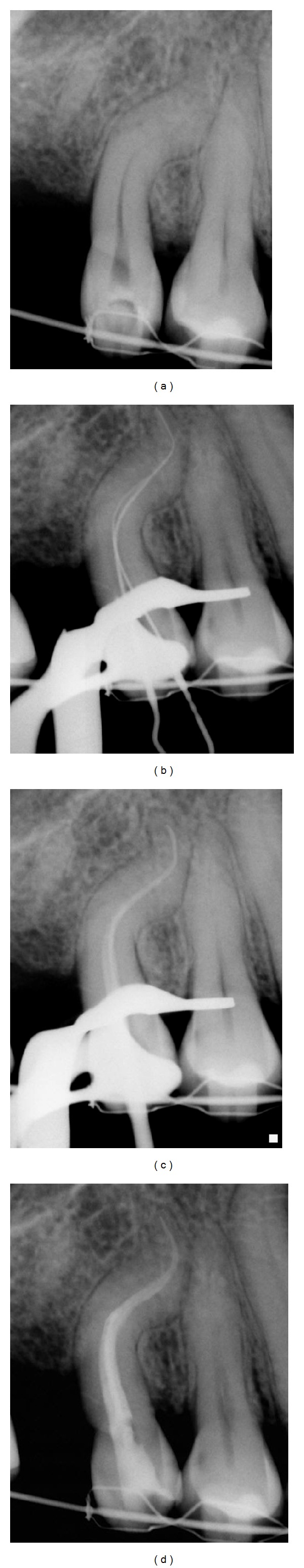
(a) Initial radiograph showing an access previously performed, (b) radiographic confirmation of the working length, (c) radiograph taken to check master cone, and (d) final radiograph.

A 37-year-old male patient was referred to the clinic of the one of the authors (Carlos Saucedo) for the endodontic treatment of his right maxillary second premolar. Treatment had been previously attempted at an endodontist's office and was incomplete. The medical history was noncontributory. As the initial access had already been accomplished, thermal tests were not performed. The adjacent teeth exhibited unremarkable findings. Probing depths were within 3 mm. Preoperative radiograph demonstrated that access had been previously performed and a double curve or S-shaped anatomy (Figure 4(a)).

After administrating infiltration anesthesia (articaine 1 : 100.000 epinephrine) the rubber dam was placed; the previous temporary material was removed by using a 1016HL bur (Dentsply Maillefer, Ballaigues, Switzerland) and refinement of the access cavity was achieved using the Endo Z bur (Dentsply Maillefer, Ballaigues, Switzerland). As only one canal had been previously found toward the buccal aspect, the access preparation was extended toward the palatal side finding the palatal canal. Initial negotiation was performed with C+ .06 K files (Dentsply Maillefer, Ballaigues, Switzerland) 3-4 mm short of the radiographic apex. Irrigation with 17% EDTA was performed and the coronal two-thirds was flared using the TF System (SybronEndo, Orange, CA, EUA) starting with the .25/.10 file entering 3-4 mm into the orifice followed with the .25/.08 proceeding to 5–7 mm depths. Next, the Mini Apex Locator (SybronEndo, Orange, CA, EUA) was used to establish the working length which was also validated using a digital radiograph (Figure 4(b)).

After achieving the working length with a C+ 10 K file (Dentsply Maillefer, Ballaigues, Switzerland) the HyflexCM System was used (Coltene-Whaledent, Allstetten, Switzerland) finishing the instrumentation with a .30/.04 file. Blocking of the canal was prevented by using multiple recapitulations with a K file size .10 (Dentsply Maillefer, Ballaigues, Switzerland) between each rotary file use while copiously irrigating with 5% NaOCl using the Endovac irrigation system (SybronEndo, Orange, CA, EUA). The canals were flooded with 17% EDTA solution for 2 minutes and the canals were dried with size .20 sterile paper points and obturation was performed with the continuous wave of condensation technique [12]. A size .30/.04 Hyflex master cone (Coltene-Whaledent, Allstetten, Switzerland) was placed to working length and fit (Figure 4(c)). A size .25/06 System B plugger was preselected (SybronEndo, Orange, CA, EUA) and was used approximately 5 mm short of the working length. Pulp Canal Sealer EWT (SybronEndo, Orange, CA, EUA) was the sealer used to coat the cone and the down pack was performed at 200°C with a number 2 Buchanan Plugger (SybronEndo, Orange, CA, EUA). The backfill was performed with the System B Cordless unit. Glass ionomer (Fuji IX GC Asia Dental Ptv Ltd, City Madhapur, Hyderabad, India) was placed in the access and the patient was referred for definitive restoration (Figure 4(d)).

## 3. Discussion

Anatomical complexities and double curvatures have been reported by several studies [15–17]. Complex root canals systems that are not cleaned and filled adequately might provide a source of persistent irritation, compromising the long-term success of the root canal therapy [18, 19].

The diagnosis and management of double curvatures, or S-shaped canals, present an endodontic challenge. Careful examination of preoperative radiographs is clinically helpful [20–22]. Based upon the dental literature and as shown in these described cases, it is suggested that knowledge of root anatomy is essential for endodontic treatment success. The different clinicians highlighted in this paper demonstrate useful and different clinical protocols serving as a guide for all phases of endodontic treatment. The important treatment strategy requires that careful attention is paid to anatomical complexities and that anatomical variations can be found in any portion of a human tooth root [23, 24]. A careful, meticulous, and thoughtful method avoids incomplete root canal preparation and failure.

There is a consensus in the literature that instrumentation in curved canals considering a great degree of curvature predisposes higher risks of accidents [25–27]. The four extremely curved cases detailed in this paper show how strategic planning can lead to successful achievement of objectives. The authors of this paper described different cleaning and shaping protocols and different techniques of warming gutta percha.

In general, endodontics is a very complex discipline and an educational challenge for those institutions teaching the specialty. Studies have shown unsatisfactory endodontic treatments with preventable errors performed by undergraduate students [28–31]. This paper provides useful clinical suggestions provided by four geographically and culturally diverse clinicians experienced in performing endodontic therapy. Their unique insights, experiences, and knowledge may help to educate dentists who would like to successfully treat complicated endodontic cases.

## 4. Conclusion

Endodontic therapy is a very nuanced and challenging science and art. The clinical and teaching experience of the authors show different approaches that can be successfully employed to treat challenging teeth having roots with multiple curves. Technical principles of endodontic treatment require constant assessment, revisions, and definition.
